# Chronic Kidney Disease in Tasmania: Protocol for a Data Linkage Study

**DOI:** 10.2196/20160

**Published:** 2020-09-17

**Authors:** Timothy Saunder, Alex Kitsos, Jan Radford, Kim Jose, Charlotte McKercher, Rajesh Raj, Nadine Wiggins, Brian Stokes, Matthew D Jose

**Affiliations:** 1 School of Medicine University of Tasmania Hobart Australia; 2 Axion Data Hobart Australia; 3 School of Medicine University of Tasmania Launceston Australia; 4 Menzies Institute for Medical Research University of Tasmania Hobart Australia; 5 Renal Unit Launceston General Hospital Launceston Australia; 6 Tasmanian Data Linkage Unit University of Tasmania Hobart Australia; 7 Renal Unit Royal Hobart Hospital Hobart Australia; 8 Australia and New Zealand Dialysis and Transplant Registry (ANZDATA) Adelaide Australia

**Keywords:** chronic kidney disease, dialysis, transplantation, data linkage.

## Abstract

**Background:**

Chronic kidney disease (CKD) is a significant and growing health burden globally. Tasmania has the highest state prevalence for non-Indigenous Australians and it has consistently had the lowest incidence and prevalence of dialysis in Australia.

**Objective:**

To examine the gap between the high community prevalence of CKD in Tasmania and the low use of dialysis.

**Methods:**

This is a retrospective cohort study using linked data from 5 health and 2 pathology data sets from the island state of Tasmania, Australia. The study population consists of any person (all ages including children) who had a blood measurement of creatinine with the included pathology providers between January 1, 2004, and December 31, 2017. This study population (N=460,737) includes within it a CKD cohort, which was detected via pathology or documentation of kidney replacement therapy (KRT; dialysis or kidney transplant). Kidney function (estimated glomerular filtration rate [eGFR]) was calculated using the Chronic Kidney Disease Epidemiology Collaboration (CKD-EPI) formula. Individuals with 2 measures of eGFR<60 mL/min/1.73 m^2^, at least 90 days apart, were identified as having CKD and were included in the CKD cohort. Individuals treated with dialysis or transplant were identified from the Australia and New Zealand Dialysis and Transplant Registry.

**Results:**

The study population consisted of 460,737 people (n=245,573 [53.30%] female, mean age 47.4 years) who were Tasmanian residents aged 18 years and older and were followed for a median of 7.8 years. During the later 5 years of the study period, 86.79% (355,622/409,729) of Tasmanian adults were represented. The CKD cohort consisted of 56,438 people (ie, 12.25% of the study population; 53.87% (30,405/56,438) female, mean age 69.9 years) followed for a median of 10.4 years with 56,039 detected via eGFR and 399 people detected via documentation of KRT. Approximately half (227,433/460,737, 49.36%) of the study population and the majority of the CKD cohort (41,448/56,438, 73.44%) had an admission episode. Of the 55,366 deaths recorded in the study population, 45.10% (24,970/55,366) had CKD.

**Conclusions:**

Whole-of-population approaches to examine CKD in the community can be achieved by data linkage. Over this 14-year period, CKD affected 12.25% (56,438/460,737) of Tasmanian adult residents and was present in 45.10% (24,970/55,366) of deaths.

**International Registered Report Identifier (IRRID):**

DERR1-10.2196/20160

## Introduction

Chronic kidney disease (CKD) is a significant and growing public health burden that manifests in substantial burden of illness and premature mortality [1]. It has been named one of the “most neglected chronic diseases” [2] and has a complex interaction with other conditions, serving as a multiplier of risk in all populations. CKD significantly increases the underlying risk of premature death, hospitalization, cancer, diabetes, or major vascular events by twofold to fivefold [2]. In 2012, the total costs attributable to CKD in Australia were estimated at AUD 4.1 billion (US $3 billion) [3]. Even early stage CKD is associated with a 50% increase in health-related expenditure; with later stages this is a sixfold increase [3]. Much of this cost is associated with the use of kidney replacement therapy (KRT; dialysis or kidney transplants).

Tasmania is the island state of Australia with a population of half a million people spread over 68,000 km^2^. The whole of Tasmania is classified as rural or remote with many people (37%) living in areas of high disadvantage. The median age is 42 years, which is 5 years older than the median for Australia [4]. A significant proportion of the Tasmanian population is known to have CKD [5] with Tasmania having the highest state prevalence of CKD (highest prevalence of estimated glomerular filtration rate [eGFR] <60 mL/min/1.73 m^2^ and the highest prevalence of albuminuria) [6] among non-Indigenous Australians. Conversely, Tasmania has the lowest incidence of uptake of dialysis and transplant (87 per million population [pmp] compared with 124 pmp nationally) and the lowest prevalence of dialysis (400 pmp compared with 536 pmp nationally) [7,8]. Currently, the reasons for this gap between high community prevalence of CKD and low use of dialysis remain unknown. Dialysis use in all Australian states is funded by the government and therefore decisions on its use are not directly influenced by cost to the dialysis patient. Despite this model, dialysis prevalence ranges from 2956 pmp in the Northern Territory down to 400 pmp in Tasmania, likely influenced by local population characteristics and treatment pathways.

Linking routinely collected health data, pathology, and registry data, we examine the gap between the high community prevalence of CKD in Tasmania and the low use of dialysis. We hypothesize that many Tasmanians with CKD will (1) have a slow decline in kidney function (eGFR), (2) experience multiple comorbidities, (3) have a higher mortality rate than those without CKD, and (4) have limited access to tertiary health services depending on their rurality and socioeconomic status.

Specific aims of this data linkage study are to (1) confirm the Tasmanian burden of CKD; (2) identify geographic, gender, and age-related variation in CKD burden, progression, and use of dialysis; (3) report the mortality rate and survival of Tasmanians with CKD; (4) examine how quickly kidney function deteriorates; (5) report the general health burden of Tasmanians with CKD including diabetes, hyperlipidemia, cancer, and cardiovascular disease.

This will give us better quantitative information about the detection and progression of CKD in Tasmanians, allowing us to better understand this gap and identify health service and community needs for better management of CKD in Tasmania.

## Methods

### Overview

This is a retrospective cohort study using linked data from 2 pathology providers and 5 administrative health data sets, from January 1, 2004, to December 31, 2017, 14 years in total, in the Australian state of Tasmania, an area of 68,400 km^2^ with approximately 510,000 people [9].

### Data Sets for Linkage

**Figure 1:**
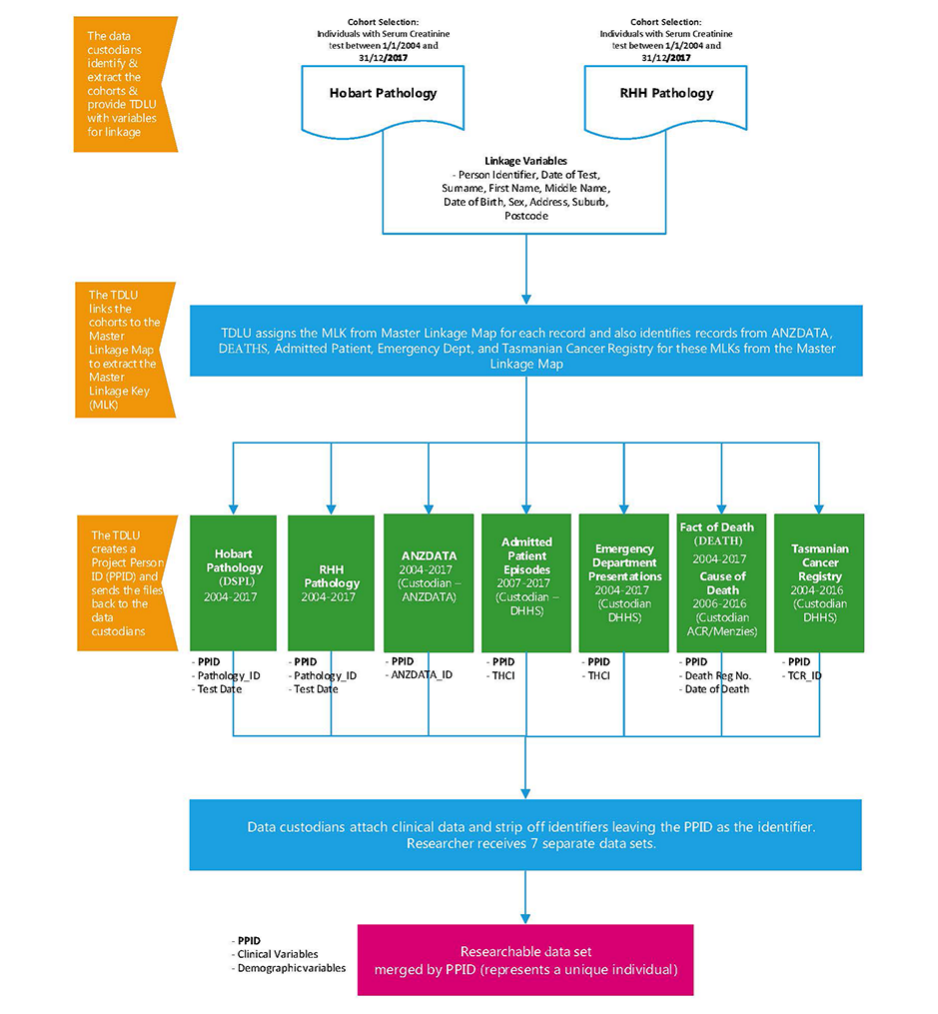
Linkage map.

Ethical approval was granted by the Tasmanian Health and Medical Human Research Ethics Committee (project H0016499, approval date June 26, 2017) to access and link the following data sets (Figure 1):

- Community and Hospital Pathology (Diagnostic Services Pty Ltd [DSPL]): Hobart Pathology, Launceston Pathology, and North-West Pathology. Pathology records provided included date, eGFR, urinary albumin-to-creatinine ratio, measures of diabetes (HbA1C), cholesterol, etc.
- Royal Hobart Hospital Pathology (RHHPATH): The largest Tasmanian public hospital pathology service provider. The data set includes pathology results for both community and hospitalized Tasmanians. Pathology records provided are the same as Community Pathology (above).
- Australia and New Zealand Dialysis and Transplant Registry (ANZDATA): records on all Tasmanians treated with dialysis or kidney transplant including the cause of kidney disease and details of their dialysis or transplant.
- Public Hospital Admitted Patient data set: date, admission, discharge, and clinical variables including primary and related diagnoses by ICD-10 code (10th edition of International Classification of Diseases and related health problems) and classification by the Australian Refined Diagnosis-Related Group for episode costing.
- Public Hospital Emergency Department Presentations: date, episode, discharge, and clinical variables including diagnoses and urgency-related groups for costing.
- Tasmanian Cancer Registry (TCR): clinical information including date, type, and stage of cancer.
- Tasmanian Fact of Death (DEATH): date of death from Births, Deaths and Marriages Tasmania; and Tasmanian Cause of Death, which presents both coded underlying and contributing cause of death from the Australian Coordinating Registry.

### Study Population

This retrospective cohort study consists of an overall study population cohort that includes within it a CKD cohort, consisting of an eGFR cohort and a KRT cohort.

The study population consists of any adult (aged 18 years and older) who had a blood measurement of creatinine with either pathology provider, RHHPATH or DSPL, between January 1, 2004, and December 31, 2017. These providers provide the majority of community pathology services to Southern, Northern, North-West, and West of Tasmania and provide inpatient pathology services to public and private hospitals in both the south and north-west. We were unable to obtain pathology results from Launceston General Hospital or smaller Tasmanian private pathology providers. We then identified people as being Tasmanian by comparing the recorded postcode on pathology report at the date of creatinine measurement against known Tasmanian Statistical Areas Level 2 (SA2).

The eGFR cohort is identified from within the study population, where a patient has 2 recorded eGFR < 60 mL/min/1.73 m^2^, at least 90 days apart [10], but no more than 3 years apart. We then added to this people identified in the KRT cohort (below) to comprise the CKD cohort.

The KRT cohort is identified from within the study population using ANZDATA [7,8]. This registry was established in 1963 and maintains records of all patients with end-stage kidney disease (ESKD) receiving dialysis or transplantation in Australia and New Zealand. All patients residing in Tasmania receiving ESKD treatment and recorded in ANZDATA between January 1, 2004, and December 31, 2017, who had blood creatinine measured in the study period from the pathology providers above, are included in the KRT cohort.

### Data Linkage

All linkage was performed by the Tasmanian Data Linkage Unit (TDLU), based at the Menzies Institute for Medical Research, University of Tasmania. The TDLU utilizes probabilistic record linkage using specialized data linkage software. This process attempts to link pairs of records based on the probability of them belonging to the same individual. The technique assigns scores based upon the agreement or disagreement of various linkage fields, and based upon the total score, record pairs are classified as either *matches* or *nonmatches*. The matches are recorded in the TDLU’s Master Linkage Map. A series of Structured Query Language (SQL) queries are executed against the Master Linkage Map to check for incorrect links (false positives) and missed links (false negatives).

The TDLU uses a combination of linkage fields including source system identifier; first name, middle name, and surname; date of birth and date of death; gender; street address; suburb; and postcode. Blocking strategies (only matched pairs that meet certain basic criteria) are used to make the linkage process more efficient. In order for two individuals to be compared, they must have matching values for one or more blocking variables. The TDLU currently blocks on surname, street address, date of birth, derived postcode/month of birth variable. A process of deduplication is applied to data sets prior to linkage. This process identifies duplicate records within one data set using specified linkage variables.

After linkage of 2 or more data sets is completed, a process of clerical review is conducted. This ensures records that reach the score threshold, and are linked, are true matches. Only those pairs of records where there is at least one mismatch, based on either first name, surname, date of birth, date of death, or sex, between the newly linked record and the remaining records in the group are reviewed. This is known as the *review pool* (pool). The first stage of clerical review is to review all the groups in the *pool* where the linkage system has combined 3 or more groups of individuals (2 or more groups from the existing Master Linkage Map and the new record being linked). This situation occurs where there are 2 or more existing groups that are potential matches for the new record. In the majority of cases, the new record will become part of an existing group. There are a small number of cases where the new record contains information that allows 2 previously distinct groups to be merged into 1 group.

The second stage of the clerical review process selects groups from the *pool* where the new record has been linked with only 1 existing group from the Master Linkage Map. Groups are reviewed based on the scores of the new record to other records within the group. Groups with low scores are clerically reviewed first to check for false positives. Reviewers then work upward through the scores until no further false positives are identified. Following this stage of review, a further round of quality assurance is conducted to ensure the linked data satisfy a range of logic checks. These checks aim to identify and correct false positives (where 2 individuals have been incorrectly linked) and false negatives (where records for the same individual have not been linked).

On successful linkage, each individual identified was assigned a project person identifier (PPID). There were 521,320 PPIDs generated during data linkage (Table 1).

**Table 1:** Linkage matrix: number of individuals identified in each data set.

	DSPL^a^ pathology^b^	RHH^c^ pathology^b^	ANZDATA^b,d^	Admitted patient^e^	Emergency department^b^	Tasmanian Cancer Registry^f^	DEATHS^b,g^
DSPL pathology^b^	*490,026* ^h^	123,191	1098	237,122	315,293	41,764	53,364
RHH pathology^b^	123,191	*154,485*	835	106,827	125,038	19,065	26,171
ANZDATA^b^	1098	835	*1214*	998	1060	184	480
Admitted patient^e^	237,122	106,827	998	*253,036*	221,297	30,535	38,234
Emergency department^b^	315,293	125,038	1060	221,297	*337,914*	31,062	46,422
Tasmanian Cancer Registry^f^	41,764	19,065	184	30,535	31,062	*42,467*	18,209
DEATHS^b^	53,364	26,171	480	38,234	46,422	18,209	*55,366*

^a^DSPL: Diagnostic Services Pty Ltd.

^b^January 1, 2004, to December 31, 2017.

^c^RHH: Royal Hobart Hospital.

^d^ANZDATA: Australia and New Zealand Dialysis and Transplant Registry.

^e^January 1, 2007, to December 31, 2017.

^f^January 1, 2004, to December 31, 2016.

^g^DEATHS: Births, Deaths and Marriages Tasmania (Fact of Death).

^h^Italics indicate where data sets align.

### Geocoding

The TDLU, in partnership with the Menzies Institute for Medical Research, has developed a geocoding module to enable the derivation of latitude and longitude for large unit record data sets. The module uses fuzzy matching to link address data to the Geocoded National Address File. After processing, the system allocates a latitude and longitude at unit record level based on the following geographic levels:

- Address: Records coded to the *Address* level have been found as an exact match in the database and matched on street number, street name, suburb, state, and postcode.
- Street: Records coded at the *Street* level have been matched on street name and suburb, but the street number could not be found. Records have been coded to the midpoint of the street.
- Locality: Records coded at the *Locality* level have been matched on suburb, state, and postcode only. Records have been coded to the midpoint of the matching suburb.
- PO Box: These represent records geocoded to the Locality of the PO Box address.
- None: These records relate to addresses that could not be found in the database or Google Maps.
- Manual: These records have been found manually in Google Maps through a clerical review module and had their latitude and longitude recorded manually.

Once a latitude and longitude have been assigned, the system assigns a *meshblock* (defined as a geographic area) code to enable structures from the Australian Statistical Geography Standard to be derived. Statistical areas ranging from SA1 to SA4, as well as other Australian Bureau of Statistics geographic structures, can be produced using this system. Given the small population size and the potential for identification, we restricted the coding to SA2 and above (population areas of >5000 people).

### Data Cleaning

Access, cleaning, and analysis of the linked data set were limited to 2 authors (AK and TS). In order to calculate eGFR from serum creatinine (SCr) results, each PPID requires documentation of gender (binary classification: male or female). The PPID gender was accepted if it was recorded consistently in each data set on every administrative record or if the majority of data sets had gender recorded as a specific gender. The PPID gender was left as *unknown* if it was recorded as unknown in all available data sets or was ambiguous between administrative data sets. If no gender could be determined, the pathology records were removed from the data set. There were 357 individuals of the total PPIDs generated during data linkage (521,320, 0.07%), with gender not recorded as male or female.

### Detection of CKD via Pathology (eGFR Cohort)

SCr result was used to calculate eGFR using the Chronic Kidney Disease Epidemiology Collaboration (CKD-EPI) equation [11,12]. Tasmanian laboratories use enzymatic assays for measurement of creatinine and all results are isotope dilution mass spectrometry aligned as previously reported [13]. Stages of CKD were classified based on first eGFR according to National Kidney Foundation's Kidney Disease Outcomes Quality Initiative (KDOQI) guidelines [10] and include stage 1 (eGFR≥90 mL/min/1.73 m^2^), stage 2 (eGFR=60-89 mL/min/1.73 m^2^), stage 3a (eGFR=45-59 mL/min/1.73 m^2^), stage 3b (eGFR=30-44 mL/min/1.73 m^2^), stage 4 (eGFR=15-29 mL/min/1.73 m^2^), and stage 5 (eGFR<15 mL/min/1.73 m^2^ or treated with dialysis or a transplant).

The following CKD-EPI formulae were used:

- For females with SCr ≤62 μmol/L: eGFR (mL/min/1.73 m^2^)=144×(SCr in μmol/L × 0.0113/0.7)^–0.329^× (0.993)^age in years^
- For females with SCr >62 μmol/L: eGFR (mL/min/1.73 m^2^)=144 × (SCr in μmol/L × 0.0113/0.7) ^–1.209^ × (0.993)^age in years^
- For males with SCr ≤80 μmol/L: eGFR (mL/min/1.73 m^2^)=141 × (SCr in μmol/L × 0.0113/0.9)^–0.411^ × (0.993)^age in years^
- For males with SCr >80 μmol/L: eGFR (mL/min/1.73 m^2^)=141 × (SCr in μmol/L × 0.0113/0.9)^–1.209^× (0.993)^age in years^

The method to identify CKD consists of the following (simplified) steps:

1. Calculation of eGFR (CKD-EPI) from SCr for each pathology result in the study population.
2. Identification of CKD-qualifying eGFR results by filtering to include only results where eGFR is <60 mL/min/1.73 m^2^
  and PPID age at result ≥18 years.
3. Calculation of the number of days between qualifying eGFR results (eGFR < 60 mL/min/1.73 m^2^ on at least two occasions).
4. Search for a qualifying eGFR result date greater than 90 days since a previous qualifying result date, but no more than 3 years since a previous qualifying result date.
5. If a second qualifying result date was detected between 90 days and 3 years, the date of the second qualifying result was deemed to be date of diagnosis.
6. PPID was identified as Tasmanian if they had at least one Tasmanian SA2 recorded in pathology.

There were 56,039 Tasmanian PPIDs diagnosed with CKD via pathology.

### Identifying CKD via ANZDATA Registry (KRT Cohort)

Although all people treated with dialysis or transplant are classified as having CKD, the pathology method described above may not identify someone who has had a successful kidney transplant and an eGFR >60 mL/min/m^2^. Therefore the ANZDATA registry was checked for PPIDs that were not detected via the pathology detection method and had at least one treatment center within Tasmania or had received a transplant.

There were 399 Tasmanian PPIDs detected via their presence within the ANZDATA registry, due to one or more of the following: (1) PPID entry being prior to start of the pathology data set (ie, before January 1, 2004); (2) PPID having insufficient pathology results within the pathology data set prior to entry to ANZDATA; (3) PPID entry to ANZDATA prior to age 18, and subsequently reaching the age of 18 in later years; and (4) PPID had a transplant and a Tasmanian SA2 coded pathology data after date of entry to ANZDATA.

### CKD Cohort

We combined the PPIDs from both methods of detection to create a CKD cohort. In addition to PPID and sex, the following variables were extracted and added to the CKD cohort data set: Age at detection, Date at detection, Date of pathology or Date of entry to ANZDATA, Method of detection, eGFR CKD-EPI at detection, CKD Stage at detection, and SA2 at detection.

For the PPIDs that were present in ANZDATA, the following additional variables were added: Age at entry to registry, Date of entry to registry, eGFR CKD-EPI at entry to registry, Date of first dialysis, Age at first dialysis, Date of first transplant (if applicable), and Age at first transplant (if applicable)

### Pathology Variables Obtained

In addition to creatinine, we obtained 25 other pathology variables (Multimedia Appendix 1). If PPIDs had an SCr test during the study period, we received all requested pathology data for them, regardless of if they had an SCr test on the same day or not.

### Identifying Cause of CKD and Comorbidities

The cause of CKD was identified (where available) from the admitted patient data set using ICD-10 codes (Multimedia Appendix 2) and ANZDATA using primary renal disease code.

Comorbidities of interest were identified using ICD-10AM codes for diabetes (ICD-10AM codes E10-14), cardiovascular disease (ICD-10AM codes I00-99), and cancer (ICD-10AM codes C00-D48). Other comorbidities were determined using both the Charlson Comorbidity Index [14] and Elixhauser Comorbidity Index [15].

### Identifying Cause of Death

Death data are recorded in multiple data sets including DEATH, TCR, Admitted Patient, and ANZDATA. DEATH (fact of death) was used as the primary data source, with additional death dates and cause of death added from additional data sets. Specific kidney disease–related cause of death was identified using the Australian Institute of Health and Welfare report, “Deaths from Chronic Kidney Disease” [16].

## Results

### Study Population

The linkage identified 521,320 people who had an SCr test in Tasmania during the 14-year study period, of whom 460,737 were determined to be Tasmanian residents (at the time of pathology test) aged 18 years or older and therefore became *the study population*.

For the 5-year period (2013-2017) there were 355,622 unique individuals aged 18 years and older included in our linked data set. As the estimated adult resident population in Tasmania was 409,729 at this time, we estimate that our study population represents approximately 86.79% of Tasmania’s resident adult population during this 5-year period (Table 2).

**Figure 2:**
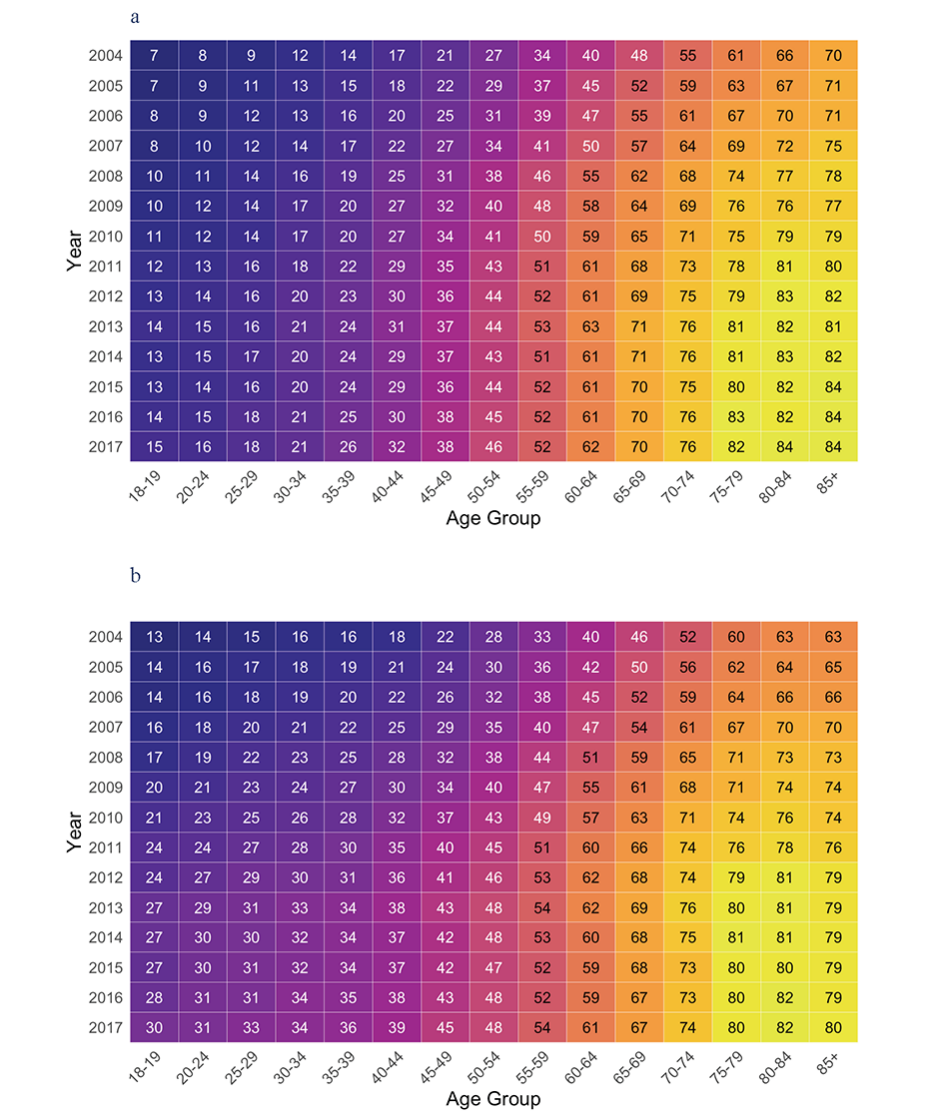
(a) Male unique PPIDs by age group per year (% of Tasmanian Population). (b) Female unique PPIDs by age group per year (% of Tasmanian Population).

The study population showed significant variation by age (Figure 2A and 2B), with the older population (aged 60 years and older) more likely to have a pathology test in this 5-year period (*P*<.001).

There were 460,737 people in the study population, of which 53.30% (245,573/460,737) were female with a mean age of 47.4 (SD 18.3) years, having 1.1 creatinine results per person per year over the 7.8 (interquartile range [IQR] 8.3) years of follow-up (Table 3).

**Table 2:** The number of unique individuals and proportion of Tasmania’s adult population included in the data set in the previous 1, 2, 3, or 5 years (% of estimated resident population [9] aged 18 years and older [ERP^a^ 18+]).

	Unique PPID^a^					Population (ERP 18+^b^)		Unique PPID % of ERP			
Year	1 year	2 years	3 years	5 years			1 year		2 years	3 years	5 years
2004	99,338	99,338	99,338	99,338	365,597		27.17		27.17	27.17	27.17
2005	109,184	157,570	157,570	157,570	369,271		29.57		42.67	42.67	42.67
2006	116,784	168,977	200,422	200,422	372,707		31.33		45.34	53.77	53.77
2007	126,803	180,495	213,435	236,465	376,512		33.68		47.94	56.69	62.80
2008	140,792	195,704	228,096	269,775	381,317		36.92		51.32	59.82	70.75
2009	149,900	209,638	242,501	284,069	386,739		38.76		54.21	62.70	73.45
2010	158,645	220,167	255,302	296,769	391,666		40.50		56.21	65.18	75.77
2011	169,235	232,304	267,756	310,200	395,228		42.82		58.78	67.75	78.49
2012	176,243	242,159	278,154	321,985	396,592		44.44		61.06	70.14	81.18
2013	183,440	250,054	287,543	332,048	397,807		46.11		62.86	72.28	83.47
2014	182,994	254,351	292,988	339,409	399,987		45.75		63.59	73.25	84.86
2015	184,173	254,519	295,896	345,153	402,512		45.76		63.23	73.51	85.75
2016	189,409	258,280	298,855	350,375	405,167		46.75		63.73	73.76	86.48
2017	195,114	265,153	304,058	355,622	409,729		47.62		64.71	74.21	86.79

^a^ERP: estimated resident population.

^b^PPID: project person identifier.

**Table 3:** Study demographics.^a^

Demographics	Study population	CKD^b^ cohort	Pathology	ANZDATA^c^
Number of individuals (18 years and older)	460,737	56,438	56,039	399
Age (years) at initial eGFR^d^, mean (SD)	47.4 (18.3)	69.7 (11.8)	69.9 (11.7)	51.2 (15.5)
Female, n/N (%)	245,573/460,737 (53.30)	30,405/56,438 (53.87)	30,267/56,039 (54.01)	150/399 (37.59)
Follow-up (years), median (IQR)	7.8 (8.3)	10.4 (6.2)	10.4 (6.2)	9.2 (9.8)
Creatinine tests per person per year, median (IQR)	1.1 (2)	2.4 (2.8)	2.4 (2.8)	9.9 (10.6)
Age (years) at death, mean (SD)	77.4 (13.9)	82.8 (9.7)	83 (9.5)	64.9 (12.5)
Number of deaths	55,366	24,970	24,777	193
PPID^e^ (%) with an admission	227,433 (49.36)	41,448 (73.44)	41,130 (73.40)	318 (79.70)
Total number of admissions	1,319,293	456,337	387,619	68,718
Admissions per person per year, median (IQR)	0.9 (4.1)	3.4 (46.8)	1.9 (24.2)	88.8 (79.3)
PPID (%) with emergency department presentation	295,943 (64.23)	44,801 (79.38)	44,441 (79.30)	360 (90.23)
Total number of emergency department presentations	1,470,819	261,603	258,343	3260
Emergency department presentations per person per year, median (IQR)	0.9 (1.5)	0.9 (1.3)	0.9 (1.3)	1.7 (2)
PPID (%) with cancer	42,099 (9.14)	12,975 (22.99)	12,906 (23.03)	69 (17.29)
eGFR (mL/min/1.73 m^2^), mean (SD)	83.9 (29.4)	56.6 (23.2)	57.1 (22.9)	33.7 (28.8)
uACR^f^ (mg/mmol), mean (SD)	13.4 (77)	26.4 (93.6)	25.5 (91.6)	50.4 (131.4)

^a^Admissions include same day and overnight.

^b^CKD: chronic kidney disease.

^c^ANZDATA: Australia and New Zealand Dialysis and Transplant Registry.

^d^eGFR: estimated glomerular filtration rate.

^e^PPID: project person identifier.

^f^uACR: urinary albumin-to-creatinine ratio.

### CKD Cohort

The CKD cohort consisted of 56,438 people, of which 53.87% (30,405/56,438) were female with a mean age of 69.7 (SD 11.8) years and median follow-up of 10.4 (IQR 6.2) years. Stage of CKD (when first meeting the criteria for CKD in the data set) was stage 3a in 73.07% (41,242/56,438), stage 3b in 20.05% (11,315/56,438), stage 4 in 5.21% (2938/56,438), and stage 5 or on KRT in 1.67% (943/56,438).

### KRT Cohort

The KRT cohort consisted of 399 people, of which there were 37.6% (150/399) of female with a mean age of 51.2 (SD 15.5) years. Follow-up of the KRT cohort was for a median of 9.2 (IQR 9.8) years.

### Admissions and Comorbidities

Of the 460,737 people in the study population, 227,233 (49.32%) were in the Admitted Patient data set for a median of 1.0 (IQR 4.6) admissions per year. Of the 56,437 people in the CKD cohort, 41,448 (73.44%) were in the admitted data set for a median of 3.4 (IQR 46.8) admissions per year (Table 3), including same-day admissions for dialysis.

### Deaths

During the 14-year study period there were 55,366 Tasmanian deaths recorded, of which 24,970 (45.10%) were in our CKD cohort. Mean age of death in our CKD cohort was 84.1 (SD 9.6) years for women and 81.3 (SD 9.6) years for men.

## Discussion

### Overview of the Protocol

The aim of this study was to take a *whole-of-population* approach to identify and follow (through data linkage) Australians who develop CKD, and identify important outcomes such as hospital admission, dialysis use, kidney transplantation, cancer, or death. We identified 460,737 individuals who had a Tasmanian residential postcode recorded at the time of pathology collection and were aged 18 years and over. Of these, 56,438 (12.25%) met the KDIGO (Kidney Disease: Improving Global Outcomes) definition of CKD [10], making this the largest Australian CKD cohort yet reported.

The strengths of this data linkage project are the whole-of-population approach (an estimated 86.79% [355,622/409,729] of resident adult population included), longitudinal nature allowing adequate follow-up (median follow-up of CKD cohort of 10.4 years), and a relatively contained island population with a low population turnover [9]. In addition, we use biochemical measures rather than self-report to determine prevalence of CKD, diabetes, and hyperlipidemia and link to legislated data sets including the Births, Deaths and Marriages Tasmania Fact of death and the TCR. Our estimates of CKD prevalence are consistent with our previously published 2007 data [5] and the Australian Health Survey [6].

The quality and completeness of the administrative data sets included were variable, but data conflicts and missingness were proportionally small (no sex recorded for 0.07% [357/521,320] of PPIDs generated during data linkage). While ethnicity was recorded in several data sets, concordance was <90% and further work needs to be done to improve recognition and recording of diversity within these administrative data sets.

Notable limitations of our study include the lack of primary health data, prescribed medications including secondary prevention strategies, general information on health risk factors including actual weight, BMI, smoking history, or blood pressure control. In addition, we rely on coding practices across public health institutions for comorbidities. Private hospital admission data were not included, nor were admissions of Tasmanian residents who travel to other jurisdictions for management of these chronic conditions.

Throughout this study we have used KDIGO definitions of CKD stating that a person with 2 measurements of eGFR <60 mL/min/1.73 m^2^ 90 days apart has CKD. This definition does not separate kidney disease from kidney aging and therefore does not take into account the call for age-specific thresholds to allow for an age-adapted definition [17].

We have commenced the analysis of this linked data set and hope to report our findings to the Tasmanian community within 12 months. The results will give us better quantitative information about detection and progression of CKD in Tasmanians, areas and populations of high disease prevalence, and risk factors for CKD. This will allow us to better understand this gap and identify health service and community needs, to optimize management of CKD in Tasmania through early detection and treatment.

### Conclusions

We have described our methodology for the largest (retrospective) Australian CKD cohort reported to date. We will use these linked data to estimate the burden of CKD in the Tasmanian community, the progression of CKD to ESKD, the presence and influence of coexisting comorbidities and important outcomes including use of KRT (dialysis or transplant), episodes of cancer, hospitalizations, and death. We will use these data to better understand the apparent gap between high community prevalence of CKD and low use of KRT.

## Appendix

Multimedia Appendix 1 Variables received from pathology services.

Multimedia Appendix 2 ICD-10AM codes used to identify the cause of CKD.

## Abbreviations

ANZDATA: Australia and New Zealand Dialysis and Transplant Registry
CKD: chronic kidney disease
DEATH: Births, Deaths and Marriages Tasmania Fact of Death
DSPL: Diagnostic Services Pty Ltd (Hobart, Launceston, and North West Pathology)
eGFR: estimated glomerular filtration rate
ESKD: end-stage kidney disease
PPID: project person identifier
RHH: Royal Hobart Hospital
RHHPATH: Royal Hobart Hospital Pathology
SA2: Statistical Areas Level 2
SCr: Serum Creatinine
TCR: Tasmanian Cancer Register
TDLU: Tasmanian Data Linkage Unit
