# Supplementary material for: Chronic Kidney Disease in Tasmania: Protocol for a Data Linkage Study
Source: JMIR Res Protoc. 2020 Sep 17;9(9):e20160. doi: 10.2196/20160 (PMC7530696; doi:10.2196/20160)
Supplement: Multimedia Appendix 1 [file resprot_v9i9e20160_app1.docx]

| **Pathology result** | **Sample** |
| --- | --- |
| Creatinine (mmol/L) | Blood |
| eGFR (mL/min/1.73m^2^) | Blood |
| Sodium (mmol/L) | Blood |
| Potassium (mmol/L) | Blood |
| Bicarbonate (mmol/L) | Blood |
| Chloride (mmol/L) | Blood |
| Calcium (mmol/L) | Blood |
| Phosphate (mmol/L) | Blood |
| Uric Acid (mmol/L) | Blood |
| Albumin (g/L) | Blood |
| Ferritin (ug/L) | Blood |
| Fasting Glucose (mmol/L) | Blood |
| Random Glucose (mmol/L) | Blood |
| HbA1c (%) | Blood |
| Vitamin D (nmol/L) | Blood |
| Triglyceride (mmol/L) | Blood |
| Cholesterol (mmol/L) | Blood |
| HDL (mmol/L) | Blood |
| LDL (mmol/L) | Blood |
| Haemoglobin (g/L) | Blood |
| ANCA | Blood |
| CRP (mg/L) | Blood |
| Urinary Albumin concentration (mg/L) | Urine |
| Urinary Creatinine concentration(mmol/L) | Urine |
| Urinary Albumin/Creatinine Ratio | Urine |
| Urinary Protein concentration (mg/L) | Urine |
