# Supplementary material for: Chronic Kidney Disease in Tasmania: Protocol for a Data Linkage Study
Source: JMIR Res Protoc. 2020 Sep 17;9(9):e20160. doi: 10.2196/20160 (PMC7530696; doi:10.2196/20160)
Supplement: Multimedia Appendix 2 [file resprot_v9i9e20160_app2.docx]

|  |  |
| --- | --- |
|  | **ICD-10 AM Codes** |
| **Chronic kidney disease** |  |
| Regular dialysis |  |
| Haemodialysis | Z49.1 |
| Peritoneal dialysis | Z49.2 |
| Other |  |
| Diabetic nephropathy | E10.2, E11.2, E13.2, E14.2 |
| Hypertensive kidney disease | I12, I13, I15.0, I15.1 |
| Glomerular diseases | N00–N07, N08 |
| Kidney tubulo-interstitial diseases | N11, N12, N14, N15, N16 |
| Chronic kidney failure | N18 |
| Unspecified kidney failure | N19 |
| Other disorders of kidney and ureter | N25–N28, N391, N392 |
| Congenital malformations | Q60–Q63 |
| Complications related to dialysis and kidney transplant | T82.4, T86.1 |
| Preparatory care for dialysis | Z49.0 |
| Kidney transplant and dialysis status | Z94.0, Z99.2 |
